# Navigation-assisted anchor insertion in shoulder arthroscopy: a validity study

**DOI:** 10.1186/s12891-020-03808-y

**Published:** 2020-12-05

**Authors:** Kyunghwa Jung, Hyojune Kim, Erica Kholinne, Dongjun Park, Hyunseok Choi, Seongpung Lee, Myung-Jin Shin, Dong-Min Kim, Jaesung Hong, Kyoung Hwan Koh, In-Ho Jeon

**Affiliations:** 1 Department of Robotics Engineering, DGIST, Daegu, Republic of Korea; 2 Department of Orthopedic Surgery, Asan Medical Center, College of Medicine, University of Ulsan, 88 Olympic-ro 43-gil, Songpa-gu, Seoul, 05505 Republic of Korea; 3 Department of Orthopedic Surgery, St. Carolus Hospital, Jakarta, Indonesia

**Keywords:** Navigation assisted, Shoulder arthroscopy, Suture anchor, Motion analysis

## Abstract

**Background:**

This study aimed to compare conventional and navigation-assisted arthroscopic rotator cuff repair in terms of anchor screw insertion.

**Methods:**

The surgical performance of five operators while using the conventional and proposed navigation-assisted systems in a phantom surgical model and cadaveric shoulders were compared. The participating operators were divided into two groups, the expert group (*n* = 3) and the novice group (*n* = 2). In the phantom model, the experimental tasks included anchor insertion in the rotator cuff footprint and sutures retrieval. A motion analysis camera system was used to track the surgeons’ hand movements. The surgical performance metric included the total path length, number of movements, and surgical duration. In cadaveric experiments, the repeatability and reproducibility of the anchor insertion angle were compared among the three experts, and the feasibility of the navigation-assisted anchor insertion was validated.

**Results:**

No significant differences in the total path length, number of movements, and time taken were found between the conventional and proposed systems in the phantom model. In cadaveric experiments, however, the clustering of the anchor insertion angle indicated that the proposed system enabled both novice and expert operators to reproducibly insert the anchor with an angle close to the predetermined target angle, resulting in an angle error of < 2° (*P* = 0.0002).

**Conclusion:**

The proposed navigation-assisted system improved the surgical performance from a novice level to an expert level. All the experts achieved high repeatability and reproducibility for anchor insertion. The navigation-assisted system may help surgeons, including those who are inexperienced, easily familiarize themselves to of suture anchors insertion in the right direction by providing better guidance for anchor orientation.

**Level of evidence:**

A retrospective study (level 2).

## Background

Although arthroscopic rotator cuff repair with suture anchor fixation is one of the most common orthopedic surgeries, it is frequently associated with suture anchor-related complications such as anchor protrusion and anchor pullout, which could result in surgical failure [11, 14]. Among the various factors that should be considered during anchor insertion, insertion location and angle are possibly the most important factors. Although insertion location is related to the microarchitecture and bone density of the anatomical footprint at the greater tuberosity [13], insertion angle is related to pullout strength when a threaded suture anchor is used.

Inserting the suture anchor at an optimum angle is essential for securing the rotator cuff at the footprint [18]. Itoi et al. showed that the threadless and threaded suture anchors resisted pullout strength at insertion angles of 45° and 90°, respectively [1–3, 5, 9].

In cases of chronic rotator cuff tear, severe degeneration of the footprint of the greater tuberosity is often found without a clear anatomical landmark. In the absence of a clear reference and due to the limitation of two-dimensional views, the placement and maintenance of the suture anchor at an optimal angle may be unreliable and irreproducible. In particular, when arthroscopic rotator cuff repair is performed by a novice operator, the lack of experience in shoulder arthroscopic surgery can increase the possibility of improper suture anchor insertion. Therefore, an improvement in the current suture anchor insertion procedure is needed to reduce errors. Micic et al. reported a navigation-assisted anchoring technique; however, the experiments were conducted only in a phantom model, with an accuracy of approximately 2° [16]. Moreover, navigation-assisted orthopedic surgical system (MAKO, Stryker®) has been used widely for knee replacements to achieve accurate alignment [19]. We hypothesized that the navigation system could be applied to anchor insertions. Therefore, this study was designed to assess the following: 1) the construct validity of the navigation-assisted surgical system to enable a novice surgeon to perform anchor insertion procedures in the phantom model and 2) the accuracy and reliability of the designated angle and location of suture anchor insertion in the humeral head for rotator cuff repair to compare the navigation and conventional techniques in cadaveric experiments. This study evaluated the following outcomes: improvements in surgical performance and accuracy of anchor insertion by the operators.

## Methods

### Navigation-assisted system for suture anchor insertion

**Fig. 1:**
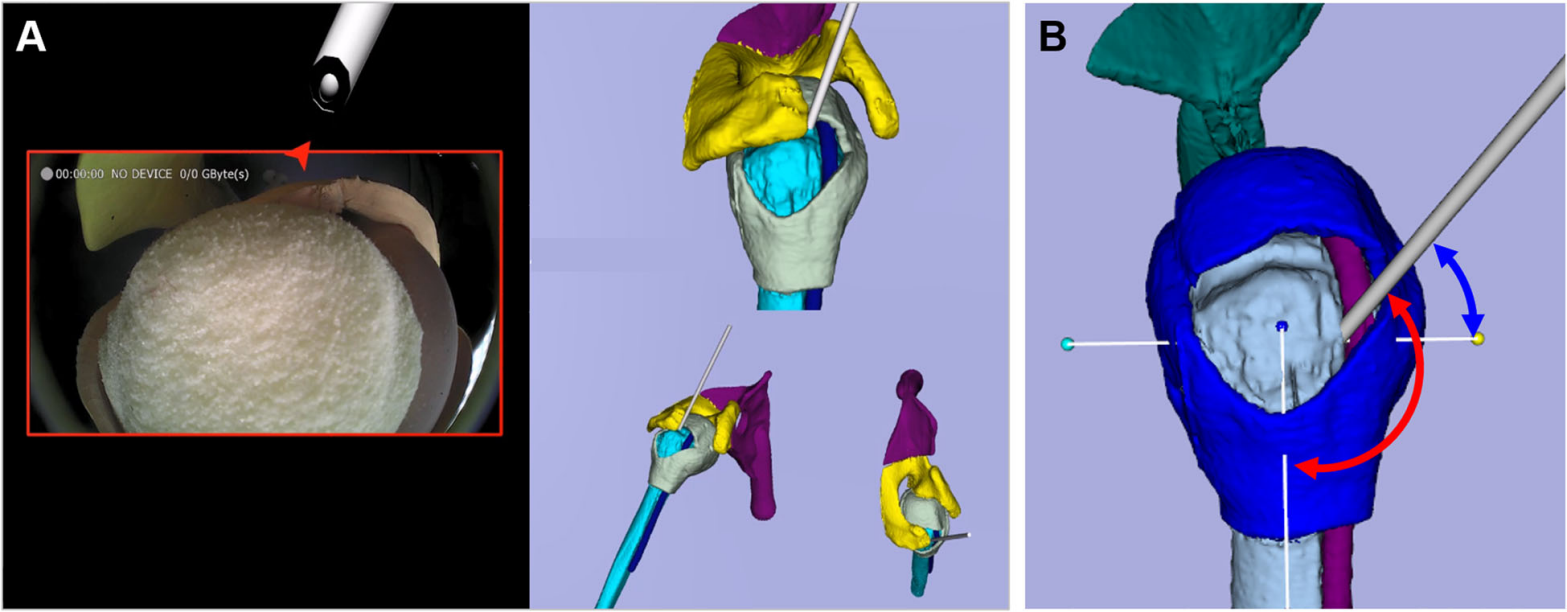
Angle-guided navigation system for a shoulder anchor insertion. **a** Screen configuration of the developed navigation system that simultaneously shows the surgical instrument and the shoulder phantom on augmented and virtual reality screens. **b** Definition of the baseline for anchor insertion angle guides

A navigation-assisted system for rotator cuff repair was developed to guide anchor insertion at an angle close to the preset target angle (Fig. 1a). This system provides both arthroscopic and navigation views. Unlike the typical arthroscopic view, the position of a surgical tool in the navigation-assisted arthroscopic view is marked by an arrow, which turns red and shows a virtual instrument when a surgical tool is not captured in the arthroscopic image or green when the surgical tool is visible in the image. This augmented reality-based technique allows the operator to keep track of the direction of surgical tools even when they are invisible on the arthroscopy screen, thereby improving the hand–eye coordination of the operator during surgery. To implement this notification function, optical tracking markers (Passive Sphere Markers, Northern Digital Inc., Waterloo, Canada) are attached to the arthroscope and surgical tools to obtain information on their location and direction and then the arthroscope camera, hand-eye, and pivot are calibrated [4].

The navigation view developed displays a three-dimensional (3D) shoulder model and surgical tools from three perspectives; this improves the 3D spatial recognition of the relative positions of the patient’s shoulder and the tool and supplements the limited depth recognition of the two-dimensional arthroscopic image. The anchor insertion angle, defined by vertical and horizontal angles, is calculated as the angle between the humerus and the surgical tool, with the vertical and horizontal baseline angles as references, respectively (Fig. 1b), and displayed in real time for the operators. The vertical baseline is a straight line that connects the greater tuberosity and surgical neck, while the horizontal baseline is perpendicular to the vertical baseline, bisecting the lesser tuberosity in a plane that faces the glenoid in front. Defining the ideal target insertion angle against each baseline before anchor insertion enables the operators to insert anchors at an insertion angle close to the target insertion angle using the proposed navigation system.

To implement the navigation view, location-tracking markers were attached to a shoulder phantom or cadaver. Cadaver-to-image registration [20] was conducted to reproduce the relative positions of the shoulder and surgical tools in a 3D virtual space. The accuracy of anchor insertion using the navigation system is determined by patient-to-image registration. The fiducial and target registration errors were 1.68 and 1.34 mm, respectively, in the phantom model and 3.76 and 3.91 mm, respectively, in the cadaveric model.

### Surgical performance evaluation using motion analysis

**Fig. 2:**
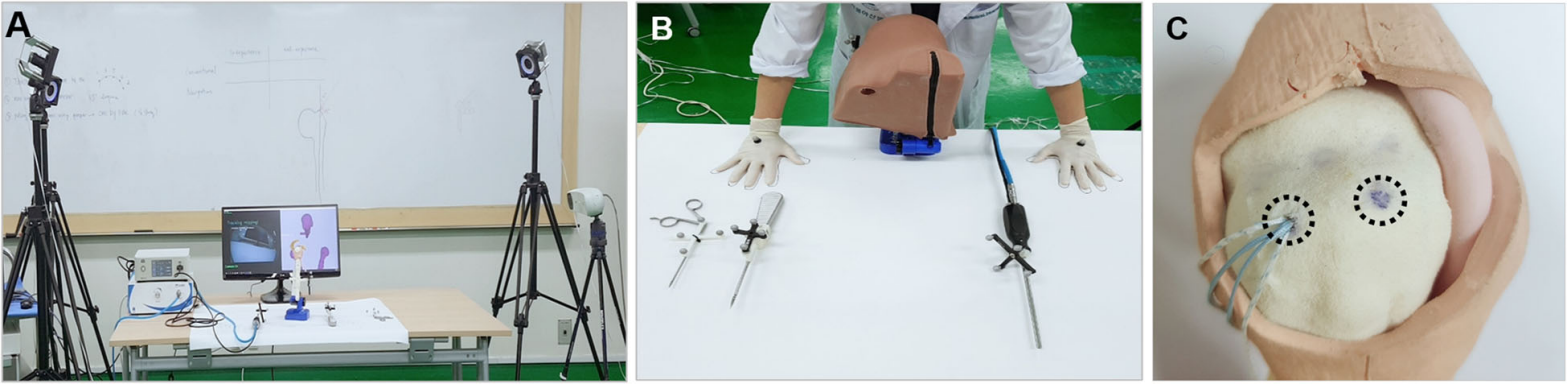
Experimental setup for motion analysis. **a** Motion analysis camera and phantom-based experimental setup. **b** Standardized positions of the arthroscopes and surgical instruments used for the anchor insertion experiment and reflective markers attached on the dorsum of the hands, arthroscope, and instruments. **c** Two anchor insertion spots on the shoulder phantom model

Surgical performance using the conventional arthroscopic system and proposed navigation-assisted system was evaluated with motion analysis (Prime 41; Natural Point, Inc., Corvallis, OR, USA; Fig. 2a). The conventional arthroscopic system (IM4000, IM4120; ConMed Linvatec, Utica, NY, USA) is a commercial 30° arthroscope measuring 4 mm in diameter with a viewing angle of 105°, whereas the proposed arthroscopic system with navigation technology (MGB Endoscopy, Seoul, South Korea) is a 0° arthroscope measuring 7 mm in diameter with a wide viewing angle of 150°. Both arthroscopic systems have an image resolution of 1920 × 1080 pixels, and the acquired images were displayed for the participating operators on a full-high-definition monitor on the experiment table.

Phantom models of the shoulder joint (Arthrex, Naples, FL, USA) were used in this study. All phantom models had rotator cuff tears of the same size. Similar experimental conditions and environment were used for all participants. The participants included three experts who were shoulder and elbow fellowship-trained orthopedic surgeons and two novices who were orthopedic residents with no experience with shoulder arthroscopy. All the five operators were right handed and handled the proposed system first and then the conventional arthroscopic system. Prior to the experiment, all participants were given instructions regarding the arthroscopic tasks and preset portals. The operators conducted a given task once with each of the two arthroscopic systems.

To ensure that the experimental environment for all participants was identical, the positions of the arthroscope, surgical tool, shoulder phantom, and both hands were preset using an arrangement tool (Fig. 2b). All participants began their tests by placing their hands on the palm contour of the arrangement tool and finished the tests by laying the arthroscope and surgical tool, as determined in advance. Two anchor insertion spots were marked, and an anchor was preinserted in one of the spots in the phantom model (Fig. 2c).

Two reflective markers were attached on the dorsal side of the third metacarpal of each operator’s hands. Four motion analysis cameras were set and calibrated prior to the experiment. The motion analysis system was capable of storing 3D (*x*, *y*, and *z*) location data with a resolution of up to 0.01 cm. Data obtained from the system were analyzed using MATLAB (R2012b; MathWorks, Torrance, CA, USA). The following surgical performance metrics were used to analyze the surgical skill of the operators: total path lengths (millimeter) of the arthroscope and surgical tool, number of movements, and time taken (seconds). The total path length refers to the sum of the distances of all 3D movements of the operator’s hands during surgery. The number of movements was defined as the number of occasions during which the instantaneous velocity exceeded the average velocity [10]. The time taken was measured from the moment when the operator inserted an arthroscope in the portal to the moment when the operator placed it on the experiment table after the completion of the given experimental task.

The experimental tasks comprised anchor insertion and anchor suture retrieval. In the former, the operator observed the arthroscopy images to find the ideal anchor insertion angle and placed the surgical tools accordingly. For the phantom models, the ideal anchor insertion angle was preset to a single value (135°) in a vertical direction. This task was performed twice at both the anchor insertion spots. The anchor suture retrieval task required the operator to pull out the four sutures of the inserted anchor from the portal one by one using an arthroscopic retriever.

### Comparison of suture anchor insertion angles in cadaveric model

**Fig. 3:**
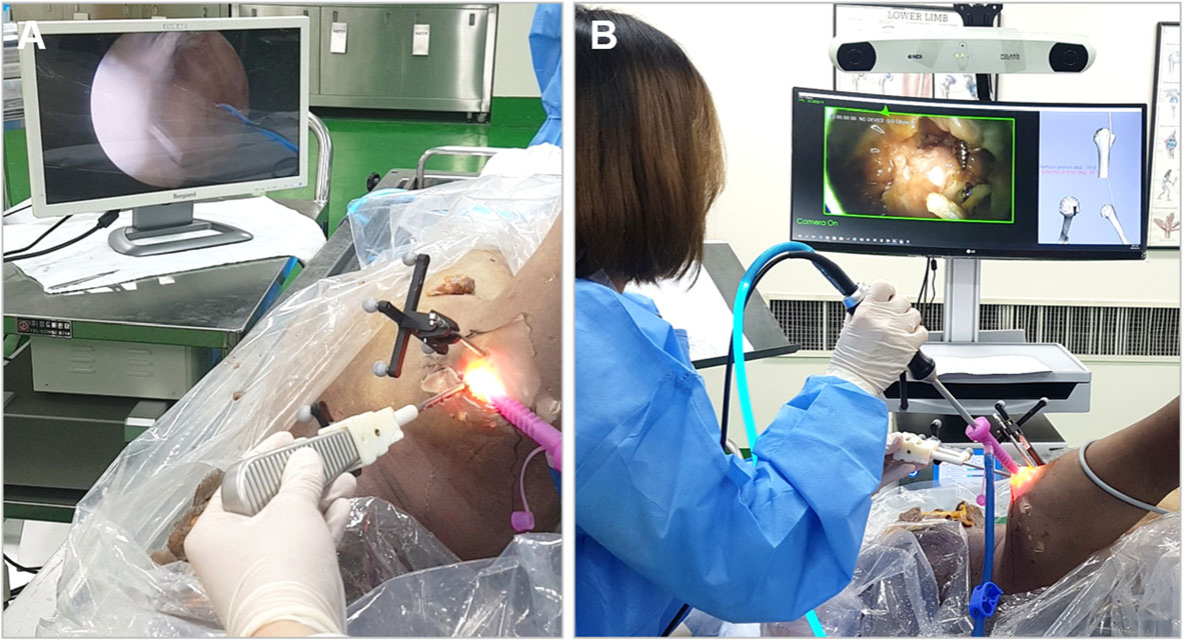
Cadaveric experiments. **a** Conventional navigation system. **b** Proposed navigation system

In cadaveric experiments, the conventional and proposed navigation-assisted arthroscopic systems were compared for variation in anchor insertion angle (Fig. 3a, b). Anchor insertion was performed three times using each system. The conventional and proposed navigation-assisted arthroscopic systems used in cadaveric experiments were the same as those used in the phantom models. Three optical tracking markers were used—a patient reference marker attached to the humerus using Steinman pins, one marker attached to the arthroscope, and one marker attached to surgical tools. To implement the position and orientation between the surgical tool and the humerus in a virtual space, patient-to-image registration was performed using anatomical landmarks, similar to that performed in the phantom experiments.

In cadaveric experiments, three participants from the experts group first performed anchor insertion using the navigation system and then the conventional system. Anchor insertion was performed three times each at the supraspinatus and infraspinatus footprints on the greater tuberosity. The ideal anchor insertion angle was set at 45°–90° from the footprint cortex by an expert surgeon with shoulder arthroscopy experience [1, 2]. Using our vertical and horizontal references, the anchor insertion angles in the vertical and horizontal directions were 140° and 90°, respectively, for the first anchor and 144° and 102°, respectively, for the second anchor. The participants performed the anchor insertion task with the goal of achieving the predetermined target angles.

As in the motion analysis experiments, the operators attempted to find the ideal anchor insertion angle and place the surgical tools accordingly. Once the operator determined the anchor insertion angle, the angle data were recorded.

### Statistical analyses

Statistical analyses of the surgical performance and anchoring angle were performed using OriginPro ver. 9b software (OriginLab Corp., Northampton, MA, USA). The data homogeneity of the three surgical performance metrics and anchoring angle errors were evaluated using the Shapiro–Wilk normality test. As all the data were not normally distributed, the paired-sample Wilcoxon signed-rank test was used to determine the statistical significance of each measurement (total path length, number of movements, and time taken) using the proposed navigation system in comparison with the conventional arthroscopic system. In addition, the Mann–Whitney *U* test was used to confirm significant differences between the expert and novice groups. The level of significance was set at *P* < 0.05, with a single asterisk indicating *P* < 0.05 and double asterisks indicating *P* < 0.01. Lastly, post-experiment power analysis was performed at a 0.05 significance level to determine the power to detect a significant difference between the two systems.

## Results

### Surgical performance evaluation using motion analysis

**Fig. 4:**
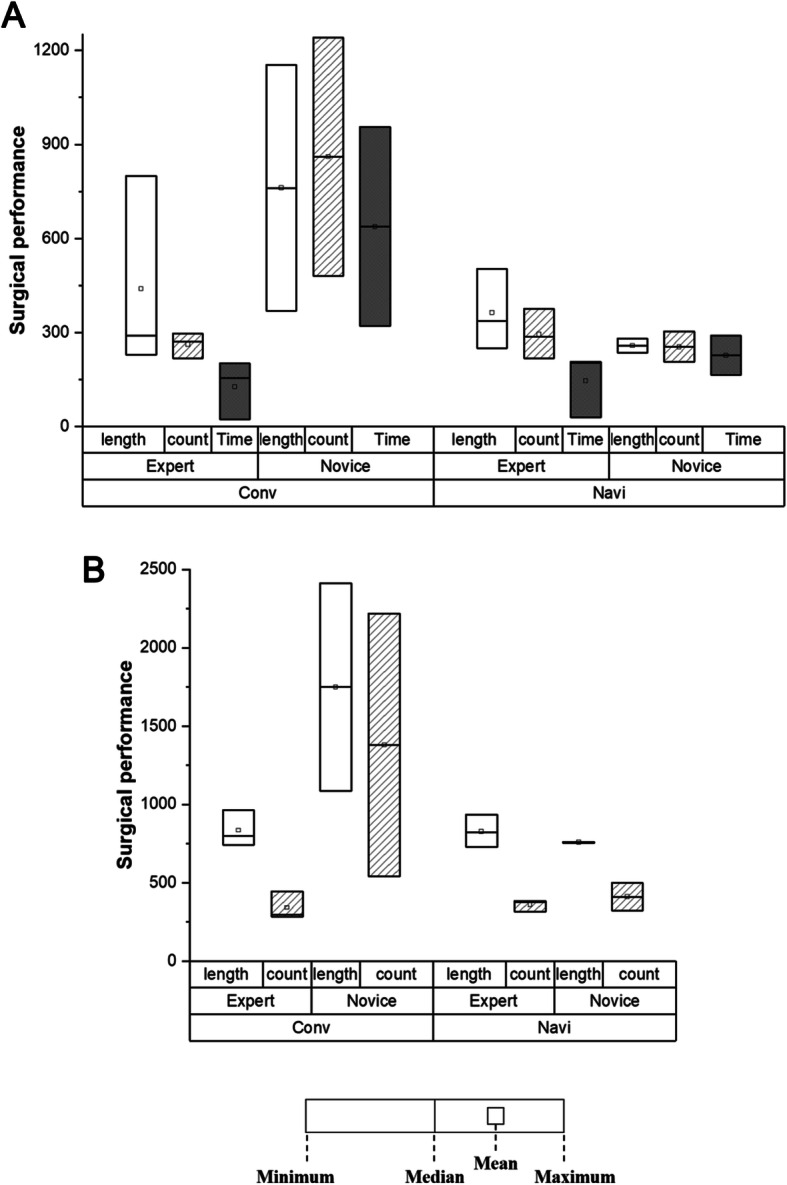
Comparison of surgical performances between experts and novices. Use of conventional (Conv) and navigation-assisted systems (Navi) for (**a**) arthroscope and (**b**) surgical instrument handling motions in the phantom model is shown. The box-and-whisker plot indicates the minimum, median, mean, and maximum values

The mean total path length of the arthroscope in the expert group decreased from 439 mm to 362.5 mm, while the mean total number of movements and time taken increased from 261.5 to 293.5 and 126 s to 145.8 s, respectively (Fig. 4a). However, no significant differences were found in these metrics. The experts handled the arthroscope similarly with both the systems, and their surgical performance with the surgical instrument was similar as well (Fig. 4b). The mean total path length of the surgical instrument decreased from 834.9 mm to 828 mm, and the number of movements increased from 340.9 to 357.6. These differences were not significant.

In the novice group, we found a difference in operating skills with both the arthroscope and surgical tool. As depicted in Fig. 4, the mean total path lengths of the arthroscope and surgical tool, number of movements, and time taken when using the proposed system were reduced. In term of surgical performance regarding arthroscope manipulation, the mean total instrument path length, number of movements, and time taken decreased from 761.1 mm to 257.5 mm, 860.4 to 254, and 637.8 s to 226.4 s, respectively. In terms of surgical performance regarding surgical instrument manipulation, the mean total instrument path length and number of movements decreased from 1748.4 mm to 757.7 mm and 1379.6 to 409.6, respectively. However, no significant differences were observed in these metrics.

**Fig. 5:**
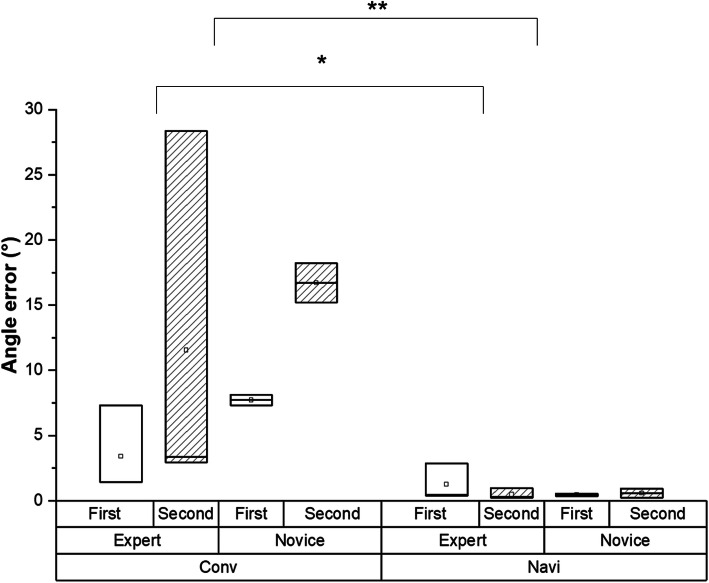
Comparison of the vertical angle errors at the first and second anchor spots. In the phantom-based anchor insertion experiments, the anchoring angle error in the expert group significantly decreased when the proposed navigation system was used (*). The mean for each participant, including those in the expert and novice groups, shows a significant difference between the conventional and proposed navigation systems in terms of anchoring angle error (**)

Further, errors in anchor insertion from the target insertion angle of 135° using the conventional arthroscopic and proposed systems at two different spots were compared (Fig. 5). The errors for the first and second anchors in the expert group, who conducted anchor insertion solely on the basis of conventional arthroscopic images, were 3.4° and 11.6° on average, respectively. These errors decreased to 1.2 ° and 0.5 °, respectively, when the proposed system was used. Similarly, the errors decreased from 7.7° and 16.7° to 0.4° and 0.6°, respectively, in the novice group. Significant differences were found between the conventional and proposed navigation systems (*P* = 0.002), and the result of the power analysis using 20 samples showed a power of 86.03%. Although the angle error significantly decreased (*P* = 0.031) in the expert group, no significant difference was found in the novice group.

### Comparison in suture anchor insertion angles in cadaveric models

**Fig. 6:**
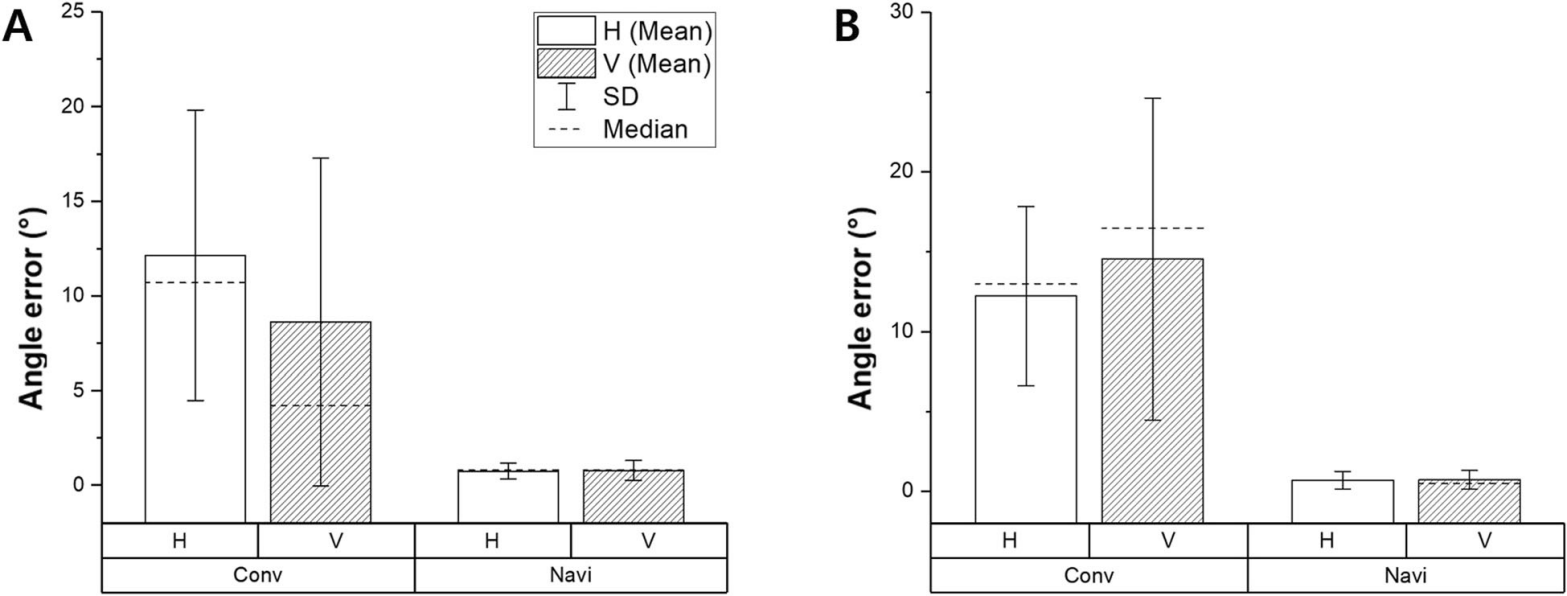
Comparison of vertical (*V*) and horizontal (*H*) angle errors in cadaver-based anchor insertion experiments. **a** First anchor. **b** Second anchors. The angle error of each expert significantly decreased when the proposed navigation system was used (*)

**Fig. 7:**
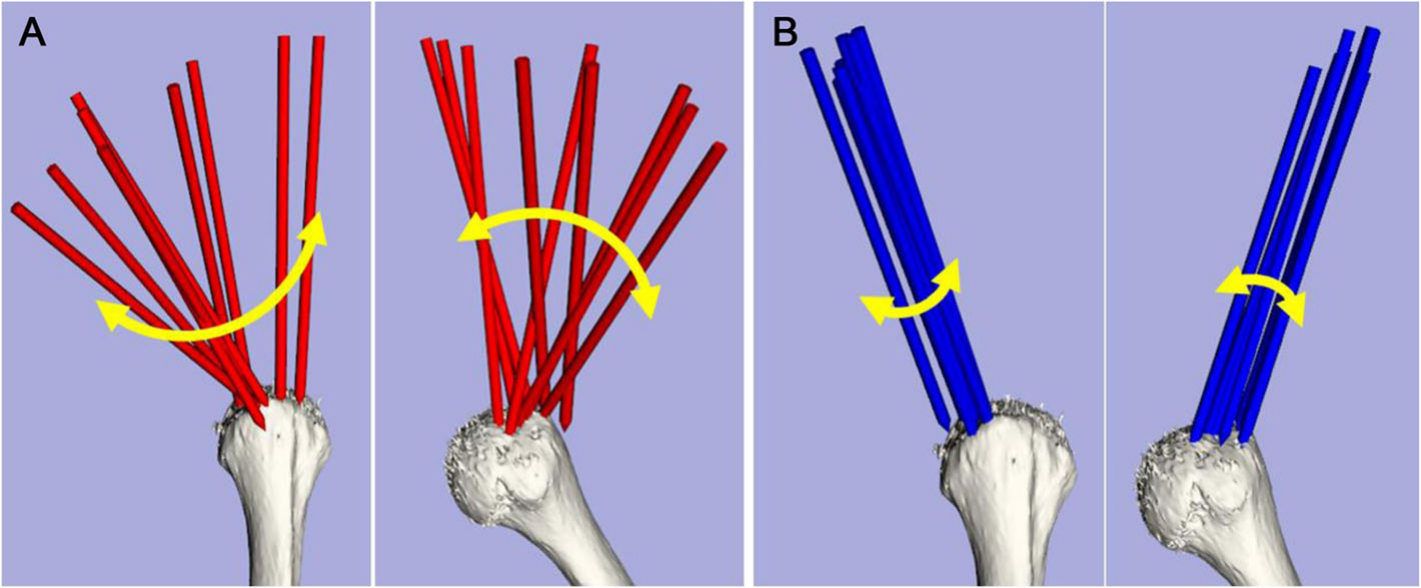
Comparison of anchor insertion angles for the first anchor. **a** Results of the conventional navigation system. **b** Results of the proposed navigation system

Anchor screw insertion angles were measured in cadaveric experiments. The results showed that the proposed navigation system reduced the deviations in the insertion angles measured when all three experts performed the three anchor insertion trials with both first and second anchors (Fig. 6). Each expert showed significant improvement (*P* = 0.031) in the vertical and horizontal angles using the proposed system, which allowed anchor insertion at an insertion angle closer to the target angle, with high reproducibility compared with the conventional system (Fig. 7). Significant improvements in the first and second anchors were powered at 99.88 and 99.99%, respectively. Furthermore, the angle deviations decreased significantly (*P* = 0.0002), not only for each expert but also among the participating experts (Table 1).

**Table 1:** Mean and standard deviation of anchor insertion angle errors in the expert group

	Anchor	Conv		Navi	
		*V*	*H*	*V*	*H*
Mean and standard deviation of the angle error (°)	First	8.6 ± 8.7	12.2 ± 7.7	**0.8** ***±*** **0.5**	**0.7** ***±*** **0.4**
	Second	14.6 ± 10.1	12.2 ± 5.6	**0.7** ***±*** **0.6**	**0.7** ***±*** **0.6**

## Discussion

The study results suggest that navigation-assisted arthroscopic rotator cuff repair could improve operators’ surgical performance, allowing anchor insertion close to the target angle in the target area. The anchor insertion angle was measured consistently in both phantom and cadaveric experiments.

The surgical skills of the operators were quantitatively evaluated using various criteria in previous studies [6–8, 10, 17]. On the basis of previous studies [12, 15], we assessed three criteria—the total path lengths of the arthroscope and surgical tool, number of movements, and operation duration. In phantom-based motion analysis experiments, the usefulness of the navigation-assisted system for surgical skill improvement varied between the expert and novice groups. However, no significant differences were found between the conventional arthroscopic and proposed systems in the three surgical performance metrics. The results indicate that the experts performed their surgical tasks while maintaining their surgical skills, regardless of the system applied. In contrast, the surgical skills of the two novices improved when the tracking and navigation systems were used. The comparison of surgical skills using the conventional system showed a large gap between the expert and novice groups, which disappeared when the navigation-assisted system was used, indicating that the surgical skills of the novices improved with the proposed system. However, no significant difference was found between groups in motion analysis experiments, which may be attributed to the small sample size.

The variation in the anchor insertion angle was narrower (< 2°) in both groups when the proposed system was used compared to when the conventional system was used. In our study, the maximum angular error of 1.2° indicated improved results, while in a previous study, a mean angular error of 2° indicated improved results [16]. Hence, the error may be within the acceptable range of anchor insertion. In addition, deviations in anchor insertion angle were narrower in the expert group than in novice group when the conventional system was used. However, the deviation in anchor insertion angle between the two groups was minimal when the proposed system was used, similar to the observations made during surgical skill evaluation. A similar trend was observed in cadaveric experiments. The surgical task performance of the novice surgeon was improved to an expert level using the proposed navigation-assistant system.

The navigation-assisted system allowed high reproducibility of anchor insertion for both the first and second anchors, with reductions in the deviations observed not only for the experts but also for the novices. As the proposed system enabled the experts to insert anchors at an insertion angle closer to the single target angle, we conclude that the proposed navigation-assisted aided the operators in inserting the anchors at a predetermined insertion angle.

By reducing the deviation of multiple anchor insertions performed by one surgeon and that between multiple surgeons, consistent, high-accuracy surgery can be performed for many patients. The advantages of the our proposed system would stand out in cases where accurately confirming the insertion angle and direction of the anchor with arthroscopy is difficult owing to the limited field of view and narrow space, especially on the glenoid side. We believe that this suggested system should be actively conducted in the future. In addition, our proposed navigation system can be used not only for the shoulder joint but also the knee, elbow, and hip joints, which require more precise and stable anchor insertion.

The present study has limitations. First, the number of participating operators was small. As the participating operators used the proposed system first and then the conventional system, the operators may have learned from the proposed system, thereby creating a ceiling effect, which may have affected their performance while using the conventional system. Second, in the phantom and cadaveric experiments, anchors were not actually fixed and surgical tools were positioned at spots determined by the operators. The possibility of any changes in the angles that might have occurred during the insertion of the anchor was not considered. Third, while the results showed significant differences between the techniques, the differences may not be significantly different from a biomechanical or clinical perspective. Fourth, for practical use of the navigation-assisted system in surgery, a less invasive or noninvasive reference marker should be attached to the patient. In addition, reliable patient-image registration should be performed to cope with the joint motion due to water circulation and shoulder traction.

## Conclusions

A navigation-assisted system was developed to guide surgeons in finding the ideal anchor insertion angle and to improve the surgical skills of less experienced operators. High reproducibility of anchor insertion, with a deviation of < 2° from the target angle, was observed when experts in arthroscopy used this system. These findings suggest that the proposed system may contribute to improved surgical performance and anchor insertion for arthroscopic rotator cuff repair.

## Data Availability

The datasets used and/or analyzed during the present study are available from the corresponding author on reasonable request.

## Abbreviation

3D: Three-dimensional
